# Drawing First Blood: Biomarkers and TAVR Outcomes∗

**DOI:** 10.1016/j.jacadv.2023.100760

**Published:** 2023-12-17

**Authors:** Craig R. Narins

**Affiliations:** Division of Cardiology, University of Rochester, Rochester, New York, USA

**Keywords:** aortic stenosis, biomarkers, transcatheter aortic valve replacement

**Figure:**
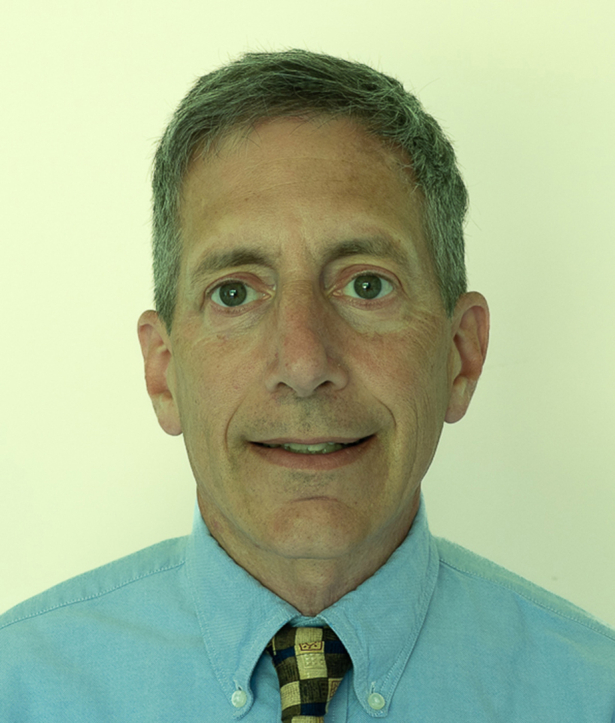

While advances in transcatheter therapy have transformed the treatment of end-stage aortic stenosis (AS) in recent years, efforts continue to better understand the optimal timing for aortic valve replacement (AVR). Current guidelines support the longstanding practice of delaying valve replacement in most instances until hemodynamically severe valve obstruction and associated symptoms are both present. Several lines of evidence indicate that prognosis for some individuals with AS begins to decline earlier in the disease process. Patients with moderate degrees of stenosis, whether symptomatic or not, appear overall to have worse clinical outcomes than those with mild or no AS.^1^ In addition, potentially irreversible cardiac “damage” affecting nonaortic valve structures frequently develops during earlier stages of AS before the degree of valvular obstruction meets the current threshold for valve replacement, and the presence of such maladaptive changes is associated with worse outcomes following AVR.^2^ This has led to speculation that earlier AVR might be of benefit in some instances, a concept currently under evaluation in randomized trials. On the other end of the continuum, more than one-third of patients with severe symptomatic AS enrolled in the pivotal high-risk transcatheter aortic valve replacement (TAVR) trials experienced treatment futility, in which they received no symptomatic improvement or died within 1 year of the procedure, suggesting that a window of opportunity for valve replacement exists.

Challenging patient groups commonly referred for consideration of AVR include those with higher gradient AS who are asymptomatic; those with low-flow, low-gradient AS in whom the risks of AVR are increased and the clinical outcomes less certain; and individuals with frailty and/or substantial comorbid conditions for whom futility of valve replacement becomes a concern. While echocardiography remains the principal modality for detecting AS and determining hemodynamic severity, identification of more sensitive indices to better individualize timing of AVR and predict postprocedure prognosis would be beneficial. Exercise testing to assess for latent symptoms in clinically asymptomatic patients and calcium scoring of the aortic valve by multidetector computed tomography have prognostic value and are recognized by guidelines as adjunctive tests that can help risk stratify patients under consideration for AVR. The potential utility of other novel parameters (cardiac magnetic resonance imaging, genetic markers, and generative artificial intelligence) in the management of AS is also of interest.^3^

As in numerous other disease states, the measurement of circulating blood biomarkers that putatively reflect the development, progression, and physiologic consequences of AS may hold promise for patient management. While our understanding of the pathobiology responsible for the inception and progression of aortic valve disease remains incomplete, endothelial damage of valve tissue with subsequent lipid deposition, inflammatory cell infiltration, and ultimately leaflet fibrosis and calcification all appear to play a role. Evaluation of circulating markers indicative of these processes may have additive value in risk stratification and patient selection for AVR. Higher levels of several blood biomarkers of inflammation, calcium metabolism, hemostasis, myocardial injury, myocardial stress and stretching, and renal function have been associated with increased rates of AS progression and a higher likelihood of adverse clinical outcomes following valve replacement.^4^

In this issue of *JACC: Advances*, Hecht et al^5^ report on a cohort of 362 patients with severe symptomatic AS who underwent TAVR at a single center. All patients had blood drawn preprocedurally for assessment of a panel of 15 biomarkers, including indices of myocardial injury and damage, inflammation, and renal function. On univariate analysis, 8 of the 15 biomarkers evaluated were significantly associated with increased mortality during clinical follow-up. Patients were stratified by the number of markers for which their blood levels were at or above median value for the whole cohort. Biomarker results correlated well with traditional predictors of risk. Patients with a greater number of elevated biomarkers were significantly older, had more cardiac and noncardiac comorbid conditions, a higher prevalence of advanced-stage heart failure, and higher Society of Thoracic Surgery and EuroSCORE II (European System for Cardiac Operative Risk Evaluation) predicted risk of mortality scores than those with fewer elevated biomarkers. The number of elevated biomarkers was also associated with echocardiographic indices of cardiac damage, including lower left ventricular ejection fraction and moderate or greater mitral and/or tricuspid regurgitation.

On multivariable analysis, the presence of a greater number of elevated biomarkers was independently associated with increased likelihoods of all-cause mortality and heart failure rehospitalization at median 2.5-year follow-up. The addition of biomarker results to clinical indices provided incremental prognostic value for the prediction of all-cause mortality. Additionally, a greater number of elevated biomarkers was independently associated with treatment futility following TAVR, defined as the occurrence of all-cause mortality, NYHA functional class III or greater heart failure, or rehospitalization for heart failure at 1 year despite TAVR.

This well-conceived and executed study supports and expands upon prior literature demonstrating associations between various blood biomarkers and prognosis among patients with AS undergoing TAVR. Some limitations do require attention. Importantly, normal reference ranges were not provided for the biomarkers assessed, and levels for individual patients were considered “elevated” simply if they were above the median value for the whole cohort. This definition differs from that used in clinical medicine, in which a lab value is not considered abnormal unless it falls outside a reference range based on results seen in 95% of a healthy population. It is therefore unknown which patients in this cohort had “elevated” biomarker levels in the traditional sense. In addition, each biomarker was given equal weight in the multimarker model, so the relative contribution of each individual marker to the predictive model is uncertain. The presence or absence of a “dose-response” effect, indicating whether incrementally higher levels of individual biomarkers were associated with increasing likelihoods of adverse outcomes, was also not reported.

In determining the clinical value of any novel marker of cardiovascular risk, an American Heart Association scientific statement recommends 6 sequential phases of evaluation.^6^ The current study examining a multimarker approach to risk stratification in AS patients undergoing TAVR fulfills the first 3 phases: 1) “proof of concept” that blood biomarker results differ among patients with and without a specified outcome, in this case adverse events following TAVR; 2) “prospective validation” of the concept in a clinical cohort; and 3) inference that the marker adds “incremental value” to established clinical variables. Additional necessary phases of evaluation before widespread use of a blood biomarker approach should be advocated include: 4) demonstration of “clinical utility” to alter individual risk sufficiently to change recommended therapy; and critically 5) substantiation of improved “clinical outcomes” when biomarker-directed therapy is tested in prospective randomized trials; and 6) “cost-effectiveness” is also a key prerequisite. Ultimately, biomarker findings are most valuable if they not only provide *risk stratification*, but if test results direct therapeutic decisions that lead to *risk modification* and improved clinical outcomes.

Regarding a potential blood multimarker approach to guide timing of TAVR, further study should also focus on determining which specific markers or combination thereof provide the best prognostic power for TAVR outcomes independent of current clinical and echocardiographic variables. The ideal biomarker assay(s) should be accurate with acceptable sensitivity and specificity for the outcome of interest, reproducible, validated across multiple study populations, widely accessible, and economical. In addition, defining and validating reference ranges with discrete cut-points to define normal and abnormal values among AS patients is crucial so that assays, if found to be of value, can be applied to clinical practice.

Blood biomarker data has become an integral part of the treatment of many diseases, ranging from the use of tumor markers in cancer detection and treatment to inflammatory markers in rheumatologic disease to lipid markers in the management of atherosclerotic disease. Along with traditional clinical and imaging parameters, the findings of Hecht et al indicate that certain blood biomarkers have prognostic relevance in AS, but proof of their value in clinical practice awaits further study. Moving forward, delineation of objective measures to help individualize management decisions and better define the “sweet spot” for performing AVR would be of great value to heart teams and the patients we serve.

## Funding support and author disclosures

The author has reported that they have no relationships relevant to the contents of this paper to disclose.
